# *Clostridioides difficile* Infections: Epidemiological and Laboratory Data from the Internal Medicine Departments of a Tertiary Care Hospital in Athens, Greece, During the Past Decade

**DOI:** 10.3390/medicina61081416

**Published:** 2025-08-05

**Authors:** Dimitris Kounatidis, Edison Jahaj, Eleni V. Geladari, Kyriaki Papachristodoulou, Fotis Panagopoulos, Georgios Marakomichelakis, Vasileios Papastamopoulos, Vasilios Sevastianos, Natalia G. Vallianou

**Affiliations:** 1 Diabetes Center, Medical School, First Propaedeutic Department of Internal Medicine, Laiko General Hospital, National and Kapodistrian University of Athens, 11527 Athens, Greece; dimitriskounatidis82@outlook.com; 2 Dermatology Department, Evangelismos General Hospital, 10676 Athens, Greece; edison.jahaj@gmail.com; 3 Third Department of Internal Medicine, Evangelismos General Hospital, 10676 Athens, Greece; elgeladari@gmail.com (E.V.G.); kyriakipap@gmail.com (K.P.); vsevastianos@gmail.com (V.S.); 4 First Department of Internal Medicine, Sismanogleio General Hospital, 15126 Athens, Greece; fotis_1992@hotmail.com; 5 Fourth Department of Internal Medicine, Evangelismos General Hospital, 10676 Athens, Greece; gmarakomichelakis@gmail.com; 6 Fifth Department of Internal Medicine and Infectious Diseases Unit, Evangelismos General Hospital, 10676 Athens, Greece; vpapastamopoulos@gmail.com

**Keywords:** *Clostridioides difficile* infections, mortality, prognostic score, hemogram-derived parameters

## Abstract

*Background and Objectives*: *Clostridioides difficile* infection (CDI) poses a major public health problem worldwide. *Materials and Methods*: In this retrospective study, we included 274 patients with CDI, who were hospitalized in Internal Medicine Departments in Evangelismos General Hospital in Athens, Greece, during the past decade. Demographic, clinical and laboratory parameters of the patients were recorded. Statistical analysis revealed an association between older age and mortality as well as heart failure and mortality among patients with CDI. *Results*: Notably, WBC (white blood count), neutrophils, NLR (neutrophil-to-lymphocyte ratio), dNLR (derived NLR), SII (systemic immune–inflammation index) and hs-CRP (high-sensitivity C-reactive protein) demonstrated a positive association with mortality, whereas serum albumin levels and PNR (platelet-to-neutrophil ratio) exhibited an inverse relationship with mortality. We propose that the aforementioned biomarkers may be used as prognostic parameters regarding mortality from CDI. *Conclusions*: Large scale studies among patients with CDI with the advent of AI (artificial intelligence) may incorporate demographic, clinical and laboratory features into prognostic scores to further characterize the global CDI threat.

## 1. Introduction

*Clostridioides difficile* infection (CDI) poses a public health issue worldwide. It is noteworthy that the CDC (Centers for Disease Control and Prevention) in the US has declared *Clostridioides difficile* as an urgent threat to public health. This is mainly due to its association with the widespread use of antibiotics, antibiotic resistance and the recurrence rates [1,2]. Despite the fact that the prevalence of CDI varies significantly among countries, the CDI rates are particularly high in the US and the EU and constitute the major cause of healthcare-associated (HA) diarrhea in these areas [1,2]. The overuse and misuse of antibiotics, the widespread use of proton pump inhibitors, the increasing rates of immunocompromised patients and individuals residing in long-term care facilities account for the higher rates of CDI [3]. Nonetheless, it should be pointed out that CDI prevalence rates are partially dependent on the quality of the surveillance systems of each country [4,5,6]. Indeed, the US has the most well-organized surveillance system as, apart from HA-CDIs, community-acquired CDIs (CA-CDIs) are also meticulously recorded [4,5,6]. Thus, differences in the reported rates of CDI prevalence across various nations may be partly attributed to differential competencies of national surveillance systems. Therefore, an improved surveillance system regarding HA-CDIs as well as CA-CDIs should be implemented to improve real estimations regarding CDIs globally.

Apart from HA-CDI, CA-CDI rates are also of considerable concern. The ECDC (European Centers for Disease Control and Prevention) has reported that HA-CDI accounted for 60.9% of cases of CDI in 2020, whereas CA-CDI accounted for 33.5% and recurrent CDI accounted for 5.6% [7]. The increasing rates of CA-CDI together with the substantial mortality of CDI, which has been associated—among other features—with the presence of binary toxin of ribotypes 027 and 078, are particularly challenging [8,9]. Early recognition of CDI together with appropriate treatment remain of paramount importance. The severity of CDI is defined by the presence of WBC > 15,000 cells/m^3^ and/or serum creatinine levels ≥ 1.5 mg/dL [10]. The severity of CDI is defined according to the American College of Gastroenterologists and determines treatment options [10]. Nevertheless, more specific and validated scores are needed to further characterize morbidity and mortality rates [11,12]. Therefore, in this study, we aimed to relate various demographic, clinical and laboratory factors that differed among patients who died from CDI in our hospital, when compared to patients that survived CDI. Our goal was mainly to find associations between laboratory parameters among survivors versus non-survivors with CDI.

## 2. Materials and Methods

Between 1 January 2015 and 31 December 2024, a total of 274 cases of CDI were included in this study among hospitalized patients in the Internal Medicine Departments of Evangelismos General Hospital, a tertiary care hospital in Athens, Greece. CDI cases were defined as patients having diarrhea (>3 watery stools daily) and a positive test for *Clostridioides difficile* toxin A or/and toxin B. In addition, we recorded characteristics such as age, sex, LOS (length of stay), outcome (survivors versus non-survivors) and comorbidities such as cancer, diabetes, heart failure, renal failure, hepatic insufficiency and respiratory failure. We measured WBC (white blood count), neutrophils, lymphocytes, monocytes, basophils, eosinophils, Ht (hematocrit), Hb (hemoglobin), PLTs (platelets), serum albumin, total protein, total and direct bilirubin, serum urea, creatinine, glucose, cardiac troponin, alanine transferase, aspartate transferase, gamma glutamyltransferase, alkaline phosphatase, lactate dehydrogenase and hs-CRP (high-sensitivity C-reactive protein). Reported laboratory data were uniformly measured during the first 24 h of the patients’ admission to the hospital. Additionally, we estimated the dNLR (derived eutrophil-to-lymphocyte ratio), the PLR (platelet-to-lymphocyte ratio), the PNR (platelet-to-neutrophil ratio), the eGFR (estimated glomerular filtration rate) as assessed by the CKD-EPI equation (Chronic Kidney Disease Epidemiology Collaboration) and finally the SII (systemic immune–inflammation index, as calculated by the formula (platelet count × neutrophil count)/lymphocyte count.

Approval from the Ethical Committee of Evangelismos General Hospital was obtained on 27 June 2025 with the protocol number 233. As this study involved human participants, the whole procedure was conducted according to the Helsinki Declaration for Medical Research in Human Participants that was released in 1975 and was revised in 2013.

## 3. Statistical Analysis

Quantitative variables with normal distribution are presented as mean ± standard deviation (SD), and quantitative variables with non-normal distribution are presented as median with interquartile range (IQR). Statistical analyses were performed using SPSS v30.0 (IBM, Armonk, NY, USA) statistical software and the R coding language and environment for statistical computing (R Foundation for Statistical Computing, Vienna, Austria). Data distribution was evaluated using the Shapiro–Wilk test. Comparisons across clinical and demographic characteristics between survivors and non-survivors were performed using the non-parametric Mann–Whitney test for data with non-normal distribution, or the chi-square test, as appropriate. As this study aims to assess the predictive value of known markers in the outcome of CDI patients, we further evaluated NLR, dNLR, SII, PNR and hs-CRP, as these variables demonstrated significant differences between survivors and non-survivors. Univariate and multivariate logistic regression models were fitted to identify which variables (age, length of stay, NLR, dNLR, SII, PNR, hs-CRP) associated with mortality. Results of the fitted models are presented as odds ratios (O.R.s) with 95% confidence intervals (C.Is). Receiver operating characteristic (ROC) curves were generated for dNLR and hs-CRP using hospital mortality as the classification factor. The results are presented as area under the curve (AUC) with 95% CI. The optimal cut-off value, with its corresponding sensitivity and specificity values, was determined based on the Youden’s index. Kaplan–Meier survival analyses were performed for both dNLR and hs-CRP. For each variable, the cohort was dichotomized into two groups (high group and low group) above and below the cut-off values, as determined by the respective ROC curves. Results from the Kaplan–Meier survival analyses are presented as mean survival time ± standard deviation. Comparisons between the high group and the low group were performed using the log-rank test. All *p*-values are two-sided; significance was set at *p* < 0.05. Statistical analysis was conducted in alignment with recent educational frameworks focusing on research-based statistical learning in medical settings [13].

## 4. Results

### 4.1. Characteristics of the Study Population

A total of 274 patients (125 male and 149 female) with verified *Clostridioides difficile* infection (CDI) were enrolled in this study, presenting a median age of 82 (73–88). Based on hospital mortality, patients were assigned to two separate subgroups, survivors (n = 234) and non-survivors (n = 40). Upon admission, demographic, clinical and laboratory data were collected and are presented in Table 1.

The two groups demonstrated significant differences across the investigated biomarkers. In comparison to non-survivors, surviving patients presented a decreased neutrophil-to-lymphocyte ratio [NLR; 5.66 (3.31–10.45) vs. 12.48 (4.97–23.35); *p* < 0.001], decreased derived neutrophil-to-lymphocyte ratio [dNLR; 3.24 (2.15–5.85) vs. 6.39 (3.48–11.70); *p* < 0.001], lower systemic immune–inflammation index [SII; 1494.00 (653.2–2923.0) vs. 1992.00 (870.10–7381.00); *p* = 0.019] and lower high-sensitivity C-reactive protein [hs-CRP; 8.20 mg/L (4.08–14.43) vs. 16.50 mg/L (8.83–24.95); *p* < 0.001]. In contrast, the platelet-to-neutrophil ratio (PNR) was significantly increased in survivors in comparison to non-survivors [28.28 (17.68–48.26) vs. 21.77 (10.81–33.18); *p* = 0.001]. The platelet-to-lymphocyte ratio (PLR) demonstrated no significant differences between groups.

### 4.2. Logistic Regression Analysis

**Figure 1:**
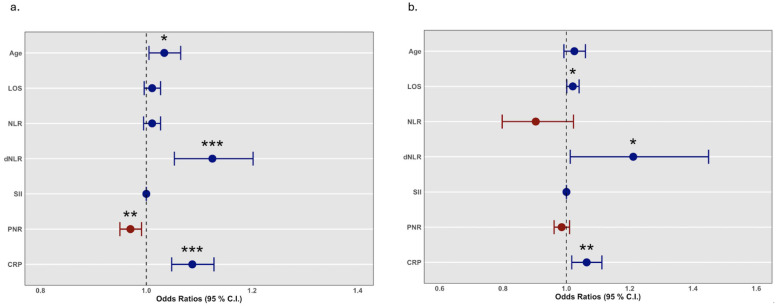
Logistic regression analysis. Forest plots of a univariate (**a**) and a multivariate (**b**) regression model of mortality predicted by age, length of stay, NLR, dNLR, SII, PNR and hs-CRP. Blue circles represent odds ratio (O.R.) values above 1, and red circles values below 1. Horizontal bars depict the 95% confidence interval (C.I.). Dashed black line: O.R. = 1.0. *, *p*-value < 0.05; **, *p*-value < 0.01; ***, *p*-value < 0.001. C.I., confidence interval; hs-CRP, high-sensitivity C-reactive protein; dNLR, derived neutrophil-to-lymphocyte ratio, LOS, length of stay; NLR, neutrophil-to-lymphocyte ratio, O.R., odds ratio; PNR, platelet-to-neutrophil ratio; SII, systemic immune–inflammation index.

Based on the results from the performed comparisons, we proceeded with biomarkers that presented significant differences between the two groups. Thus, logistic regression analysis was employed to investigate whether age, length of stay (LOS), and the biomarkers NLR, dNLR, SII, PNR and hs-CRP were associated with mortality. The univariate model demonstrated that increased age, dNLR and hs-CRP correlated with increased mortality risk, while increased PNR correlated with lower mortality risk (Figure 1a and Table 2). Next, a multivariate model was fitted to adjust results for confounding factors. As seen in Table 2, increased dNLR, hs-CRP and length of stay were positively associated with higher mortality risk (Figure 1b).

Data are presented as mean ± SD or median with interquartile range (IQR). Statistical analyses were performed using SPSS v30.0 (IBM, Armonk, NY, USA) statistical software and the R coding language and environment for statistical computing (R Foundation for Statistical Computing, Vienna, Austria). Initially, comparisons across clinical and demographic characteristics between survivors and non-survivors were performed using the non-parametric Mann–Whitney test, or the chi-square test, as appropriate. As this study aims to assess the predictive value of known markers in the outcome of CDI patients, we further evaluated NLR, dNLR, SII, PNR and hs-CRP, as these variables demonstrated significant differences between survivors and non-survivors. Univariate and multivariate logistic regression models were fitted to identify which variables (age, LOS, NLR, dNLR, SII, PNR, hs-CRP) were associated with mortality. Results of the fitted models are presented as odds ratios (O.R.s) with 95% confidence intervals (C.Is). Receiver operating characteristic (ROC) curves were generated for dNLR and hs-CRP using hospital mortality as the classification factor. The results are presented as area under the curve (AUC) with 95% CI. The optimal cut-off value, with its corresponding sensitivity and specificity values, was determined based on the Youden’s index. Kaplan–Meier survival analyses were performed for both dNLR and hs-CRP. For each variable, the cohort was dichotomized into two groups (high group and low group) above and below the cut-off values, as determined by the respective ROC curves. Results from the Kaplan–Meier survival analyses are presented as mean survival time ± standard deviation. Comparisons between the high group and the low group were performed using the log-rank test. All *p*-values are two-sided; significance was set at *p* < 0.05.

### 4.3. Receiver Operating Characteristic (ROC) Curves

**Figure 2:**
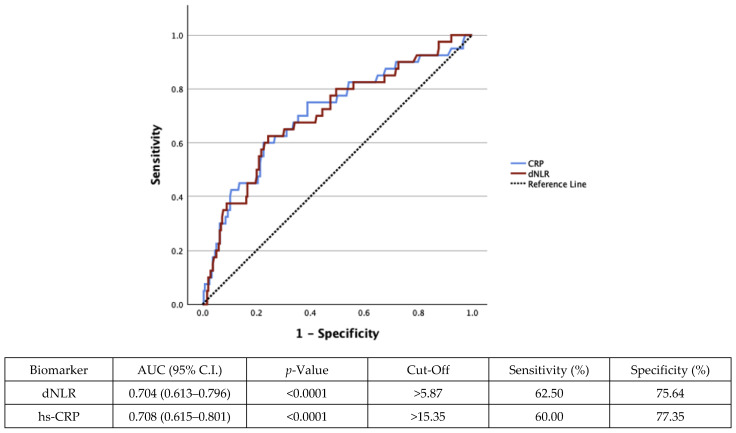
dNLR and hs-CRP as classifiers for mortality. Receiver operating characteristic (ROC) curves were generated for dNLR (red line) and hs-CRP (blue line), presenting the prognostic value of both markers for mortality. dNLR demonstrated an AUC of 0.704 (0.613–0.796), *p* < 0.0001, at a cut-off value of 5.87 based on Youden’s Index, with 62.50% sensitivity and 75.64% specificity. The calculated AUC for hs-CRP was 0.708 (0.615–0.801), *p* < 0.0001, at a cut-off value of 15.35 mg/L, with a sensitivity of 60% and a specificity of 77.35%. AUC, area under the curve; C.I., confidence interval; hs-CRP, high-sensitivity C-reactive protein; dNLR, derived neutrophil-to-lymphocyte ratio.

Regression analysis highlighted a significant association between dNLR and hs-CRP with mortality. Receiver operating characteristic (ROC) curves were generated for both dNLR and hs-CRP (Figure 2). dNLR demonstrated an area under the curve (AUC) equal to 0.704 (95% C.I.: 0.613–0.796, *p* < 0.0001), with a cut-off at 5.87, corresponding to a sensitivity of 62.50% and a specificity of 75.64%. The AUC for hs-CRP was 0.708 (0.615–0.801) (*p* < 0.0001), with a cut-off for hs-CRP at 15.35 mg/L, demonstrating 60% sensitivity and 77.35% specificity.

### 4.4. Survival Analysis

**Figure 3:**
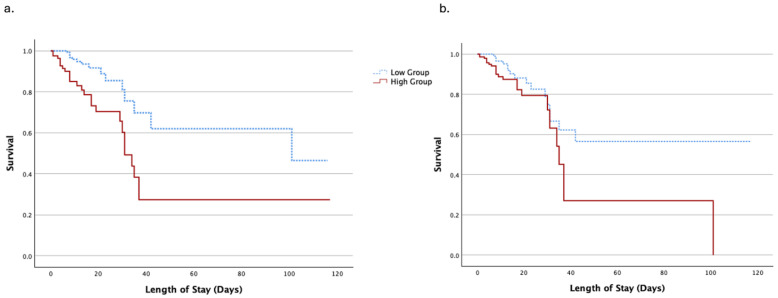
Association of dNLR and hs-CRP values with mortality probability. Kaplan–Meier analyses were performed to estimate the probability for mortality in correlation to dNLR (**a**) and hs-CRP (**b**), and in both cases the log-rank test for two-group comparison was used. (**a**) Kaplan–Meier analyses regarding dNLR. The cohort was dichotomized above and below the cut-off value (5.87; high-dNLR group and low-dNLR group) as determined by the ROC curve for dNLR. The high-dNLR group is depicted with the solid red line and the blue dashed line refers to the low-dNLR group. Mean survival time for the high-dNLR group was 49 ± 9 days, and for the low-dNLR group, it was 80 ± 9 days (log-rank test, *p* < 0.001). The high-dNLR group demonstrated 50% survival on day 31, whereas the low-dNLR group did so on day 101. (**b**) Kaplan–Meier analyses regarding hs-CRP. As previously, the cohort was dichotomized above and below the cut-off value (15.35 mg/L; high-hs-CRP group and low-hs-CRP group) as determined by the ROC curve for hs-CRP. The high-hs-CRP group is depicted with the red solid line and the blue dashed line is used for the low-hs-CRP group. Mean survival time for the high-hs-CRP group was 47 ± 9 days, and for the low-hs-CRP group, it was 78 ± 8 days (log-rank test, *p* = 0.022). The high-hs-CRP group reached a 50% survival rate on day 35, while survival in the low-hs-CRP group did not drop below 50%. On day 42, survival in the low-hs-CRP group reached 56%. hs-CRP, high-sensitivity C-reactive protein; dNLR, derived neutrophil-to-lymphocyte ratio.

Furthermore, Kaplan–Meier analyses were performed for both dNLR and hs-CRP. The cohort was dichotomized above (high group) and below (low group) the cut-off value determined from the ROC curves of both dNLR (5.87) and hs-CRP (15.35 mg/L) using the Youden index (Figure 3). Regarding dNLR, patients in the high group presented with a mean survival time of 49 ± 9 days, in contrast to patients in the low group, who had a mean survival time of 80 ± 9 days (log-rank test, *p* < 0.001; Figure 3a). For hs-CRP, the mean survival time for the high group was 47 ± 9 days, while the mean survival time for the low-hs-CRP group was 78 ± 8 days (log-rank test, *p* = 0.022; Figure 3b).

## 5. Discussion

The results of this study were supportive of a clear association between NLR, dNLR, SII, hs-CRP and mortality. In sharp contrast, PNR and serum albumin levels showed an inverse relationship between mortality risk among patients with CDI enrolled in this study.

It is noteworthy that most studies regarding CDI and mortality have associated demographic and clinical aspects with mortality rates [14,15,16,17]. More specifically, many studies have documented a relationship between underlying medical conditions, such as cancer, inflammatory bowel diseases, cardiovascular disease (stroke and heart failure), chronic kidney disease and use of corticosteroids with a greater risk of mortality in patients with CDI [14,15,16,17]. In addition, older age and the ongoing administration of antibiotics have also been implicated in higher mortality rates [14,15,16,17]. In our study, only older age as well as the existence of heart failure showed a statistically significant association with increased mortality rates. However, cancer, chronic kidney disease, hepatic insufficiency, respiratory failure and renal failure did not exhibit any statistically significant relationship with increased mortality.

In our study, apart from demographic data and comorbidities, we also focused upon laboratory parameters, especially hemogram-derived ones that proved to be particularly useful in the prognosis of CDI. It is noteworthy that the aforementioned parameters had been extensively studied during the COVID-19 pandemic [18,19,20,21,22]. More specifically, Asaduzzaman et al. had documented that estimations of NLR, dNLR and NPR on admission were valuable biomarkers of mortality among patients with COVID-19 [19]. Additionally, Velazquez et al. had demonstrated that NLR, NPR, PLR and SII were prognostic markers of admission to the ICU among COVID-19 patients [22]. Ghobadi et al. had found that the aforementioned parameters were significantly higher among non-survivors, when compared to survivors from COVID-19 [23]. Thus, based on these studies that were performed during the COVID-19 pandemic, we sought to explore any potential associations between hemogram-derived parameters and mortality from CDI. Indeed, we demonstrated a relationship between NLR, dNLR as well as SII and mortality from CDI.

Barbosa-Martins et al. have also documented that among patients with CDI, mortality rates were higher in patients with an increased NLR [24]. Moreover, Scarlata et al. have demonstrated that NLR, dNLR, SII and PNR were associated with increased mortality rates [18]. Our findings are in accordance with Barbosa-Martins et al. and with Scarlata et al. with the exception of PNR. In our study, we found an inverse relationship with PNR among non-survivors when compared to survivors from CDI. Further studies are needed to confirm or refute the association of PNR with mortality from CDI. Nevertheless, it seems likely that hemogram-derived parameters exhibit a prognostic potential regarding mortality rates among patients with CDI. Besides, hemogram-derived parameters are easy to be determined and almost ubiquitously available. For the aforementioned reasons, these hemogram-derived ratios could be used in everyday practice to aid clinicians in assessing mortality risk among patients with CDI. It is noteworthy that these hemogram-derived ratios are suggested to provide prognostic information regarding other gastrointestinal infections, such as acute diverticulitis [25]. Apart from hemogram-derived parameters, hs-CRP levels together with serum protein levels were correlated with increased mortality. In sharp contrast, serum albumin levels were lower among non-survivors, when compared to patients who survived from CDI. This inverse relationship between serum albumin levels and mortality from CDI has also been demonstrated by Bloomfield et al., in their systematic review [26]. Moreover, Asaoka et al. have recently reported that low serum albumin levels and increased WBC were predictive of the severity of CDI [27]. Low serum albumin levels have long been associated with the presence and prognosis of acute infection [28,29]. Indeed, infection is suggested to be the most common cause of acute hypoalbuminemia [30]. Thus, our finding of hypoalbuminemia among non-survivors from CDI is not surprising in this context.

As the mortality rates from CDI vary from 9% to 38%, and as HA-CDI is the first cause of HA diarrhea in developed countries, it is of the utmost importance to determine prognostic factors regarding CDI [31,32,33,34,35,36]. Indeed, the combination of clinical factors with hemogram-derived parameters and biochemical markers could be very challenging in this context. The advent of artificial intelligence (AI) and machine learning methods could contribute to the determination of prognostic scores that would be accurate and helpful in the prognosis of CDI [31,32,33,34,35,36]. Although AI has some drawbacks, such as ethical issues and plausible dissemination of misleading information, it should be implemented in clinical practice as it may sense even subtle variations in several values. Thus, it may serve as a valuable tool in predicting mortality among patients with CDI.

The main limitation of our study is its retrospective character that could imply the presence of bias. In addition, the single-center design of our study together with the inclusion of patients only from Internal Medicine wards are potential limitations as well. Another limitation is that we lack information regarding patients with inflammatory bowel disease and CDI, who are prone to CDI, as these patients are hospitalized in the Gastroenterology Departments. These limitations of our study could compromise the further application of our results to other subgroup populations. For the aforementioned reasons, these findings cannot be simply applied to other subpopulations without any further research. However, this was a study involving 274 patients with CDI during the past decade in Internal Medicine Departments in one of the largest hospitals in Athens, Greece.

## 6. Conclusions

In this study, we have demonstrated that increased WBC, neutrophils, NLR, dNLR, SII and hs-CRP had a positive association with mortality among patients hospitalized for CDI in Evangelismos General Hospital, Athens, Greece. On the other hand, we documented an inverse relationship between serum albumin levels, PNR and mortality from CDI. As CDI has been declared a public health threat, further studies are mandatory to improve prognostic factors regarding mortality rates. In this context, large-scale studies incorporating clinical features, hemogram-derived parameters and biochemical factors into prognostic scores with the advent of AI are eagerly anticipated.

## Figures and Tables

**Table 1:** Demographic characteristics and laboratory data of surviving and non-surviving patients with verified CDI.

	Survivors (n = 234)	Non-Survivors (n = 40)	*p*-Value
Age (years), (mean ± SD)	81 (72–87)	86 (76–92)	0.026 *
Sex, n (%)			0.733
Male	108 (46.2)	17 (42.5)	
Female	126 (53.8)	23 (57.5)	
Comorbidities, n (%)	138 (59.0)	28 (70.0)	0.222
Cancer, n (%)	47 (20.2)	8 (20.0)	>0.999
Diabetes, n (%)	62 (26.5)	14 (35.0)	0.339
Heart failure, n (%)	37 (15.9)	17 (42.5)	<0.001 ***
Liver insufficiency, n (%)	10 (4.3)	3 (7.5)	0.414
Renal failure, n (%)	46 (19.7)	8 (20.0)	>0.999
Respiratory failure, n (%)	28 (12.1)	8 (20.0)	0.205
Length of stay (median, IQR)	10 (6–17)	14 (8–31)	0.045 *
Laboratory data (median, IQR)			
White blood cells (10^3^/μL)	11.00 (6.87–14.31)	12.79 (9.10–22.55)	0.012 *
Neutrophils (10^3^/μL)	8.33 (5.07–12.05)	10.06 (7.39–20.81)	0.004 **
Lymphocytes (10^3^/μL)	1.29 (0.90–1.80)	1.17 (0.82–1.41)	0.078
Monocytes (10^3^/μL)	0.63 (0.45–0.94)	0.54 (0.41–0.97)	0.345
Eosinophiles (10^3^/μL)	0.06 (0.02–0.13)	0.04 (0.01–0.16)	0.184
Basophiles (10^3^/μL)	0.03 (0.02–0.05)	0.04 (0.01–0.08)	0.457
Platelets (10^3^/μL)	252.50 (178.80–323.50)	255.00 (156.30–360.80)	0.695
Hematocrit (HCT) (%)	32.35 (28.68–36.50)	30.20 (25.20–34.38)	0.024 *
Hemoglobin (Hgb) (g/dL)	10.80 (9.30–12.00)	10.05 (8.50–11.48)	0.012 *
NLR	5.66 (3.31–10.45)	12.48 (4.97–23.35)	<0.001 ***
dNLR	3.24 (2.15–5.85)	6.39 (3.48–11.70)	<0.001 ***
SII	1494.00 (653.2–2923)	1992.00 (870.1–7381)	0.019 *
PNR	28.28 (17.68–48.26)	21.77 (10.81–33.18)	0.001 **
PLR	188.20 (117.20–270.10)	193.3 (139.3–342.4)	0.359
hs-CRP (mg/L)	8.20 (4.08–14.43)	16.50 (8.83–24.95)	<0.001 ***
Albumin (g/dL)	3.20 (2.90–3.60)	2.50 (2.00–2.90)	<0.001 ***
Total bilirubin (mg/dL)	0.52 (0.39–0.75)	0.51 (0.33–0.71)	0.301
Direct bilirubin (mg/dL)	0.23 (0.17–0.35)	0.27 (0.17–0.39)	0.582
Total protein (g/dL)	5.80 (5.30–6.26)	5.20 (4.70–5.80)	<0.001 ***
Glucose (mg/dL)	96.00 (78.75–117.00)	100.50 (80.00–135.00)	0.538
Troponin (ng/mL)	32.00 (14.00–63.50)	47.00 (17.25–100.50)	0.353
Urea (mg/dL)	38.00 (25.00–65.75)	57.50 (29.75–103.30)	0.023 *
Creatinine (mg/dL)	0.99 (0.70–1.50)	0.95 (0.60–2.00)	0.830
Aspartate aminotransferase (U/L)	18.00 (13.50–27.00)	16.00 (13.00–28.50)	0.379
Alanine transaminase (U/L)	12.00 (8.00–20.00)	10.00 (6.00–20.00)	0.137
Alkaline phosphatase (U/L)	75.50 (56.00–97.75)	91.50 (67.00–134.00)	0.007 **
Gamma-glutamyltransferase (U/L)	21.00 (13.00–34.00)	19.00 (12.25–50.00)	0.583
Lactate dehydrogenase (U/L)	211.00 (167.00–265.00)	250.50 (206.80–322.80)	0.005 **
Creatine phosphokinase (U/L)	50.50 (27.00–94.75)	35.00 (23.00–74.00)	0.170
eGFR (ml/min/1.73 m^2^)	71.00 (36.00–94.00)	70.50 (30.00–93.75)	0.654

Data in the table are presented as individual numbers, n (%), and median with interquartile range (IQR). Patients enrolled to the study were assigned based on hospital mortality to one of two groups, survivors, and non-survivors. Statistical analysis between the two groups was performed using the non-parametric Mann–Whitney test or the chi-square test, as appropriate. Laboratory data were measured within 24 h of admission. hs-CRP, high-sensitivity C-reactive protein; dNLR, derived neutrophil-to-lymphocyte ratio, eGFR, estimated glomerular filtration rate; NLR, neutrophil-to-lymphocyte ratio; PLR, platelet-to-lymphocyte ratio; PNR, platelet-to-neutrophil ratio; SII, systemic immune–inflammation index. *, *p*-value < 0.05; **, *p*-value < 0.01; ***, *p*-value < 0.001.

**Table 2:** Univariate and multivariate logistic regression model.

Variable	Univariate Model			Multivariate Model		
	O.R.	95% C.I.	*p*-Value	O.R.	95% C.I.	*p*-Value
Age	1.034	1.005–1.065	0.023 *	1.025	0.992–1.060	0.141
LOS	1.011	0.996–1.027	0.162	1.020	1.001–1.040	0.038 *
NLR	1.011	0.995–1.027	0.176	0.903	0.797–1.022	0.108
dNLR	1.125	1.053–1.202	<0.001 ***	1.211	1.012–1.449	0.036 *
SII	1.000	1.000–1.000	0.230	1.000	1.000–1.000	0.289
PNR	0.970	0.950–0.991	0.004 **	0.985	0.961–1.010	0.234
hs-CRP	1.087	1.048–1.128	<0.001 ***	1.064	1.017–1.112	0.007 **

A univariate logistic regression analysis was fitted to analyze the relationship of age, length of stay, NLR, dNLR, SII, PNR and hs-CRP with hospital mortality. Multivariate logistic regression analysis adjusted for confounders demonstrating dNLR and CRP as independent indicators for mortality in patients with verified *Clostridioides difficile* infection. hs-CRP, high-sensitivity C-reactive protein; dNLR, derived neutrophil-to-lymphocyte ratio, LOS, length of stay; NLR, neutrophil-to-lymphocyte ratio, O.R., odds ratio; PNR, platelet-to-neutrophil ratio; SII, systemic immune–inflammation index. *, *p*-value < 0.05; **, *p*-value < 0.01; ***, *p*-value < 0.001.

## Data Availability

The original contributions presented in this study are included in the article. Further inquiries can be directed to the corresponding author.
